# A Methodology for Adaptable and Robust Ecosystem Services Assessment

**DOI:** 10.1371/journal.pone.0091001

**Published:** 2014-03-13

**Authors:** Ferdinando Villa, Kenneth J. Bagstad, Brian Voigt, Gary W. Johnson, Rosimeiry Portela, Miroslav Honzák, David Batker

**Affiliations:** 1 Basque Centre for Climate Change (BC3), IKERBASQUE, Basque Foundation for Science, Bilbao, Bizkaia, Spain; 2 Geosciences & Environmental Change Science Center, U.S. Geological Survey, Denver, Colorado, United States of America; 3 Gund Institute for Ecological Economics, Rubenstein School of Environment and Natural Resources, University of Vermont, Burlington, Vermont, United States of America; 4 Department of Computer Science, University of Vermont, Burlington, Vermont, United States of America; 5 Conservation International, Arlington, Virginia, United States of America; 6 Earth Economics, Tacoma, Washington, United States of America; University of Lleida, Spain

## Abstract

Ecosystem Services (ES) are an established conceptual framework for attributing value to the benefits that nature provides to humans. As the promise of robust ES-driven management is put to the test, shortcomings in our ability to accurately measure, map, and value ES have surfaced. On the research side, mainstream methods for ES assessment still fall short of addressing the complex, multi-scale biophysical and socioeconomic dynamics inherent in ES provision, flow, and use. On the practitioner side, application of methods remains onerous due to data and model parameterization requirements. Further, it is increasingly clear that the dominant “one model fits all” paradigm is often ill-suited to address the diversity of real-world management situations that exist across the broad spectrum of coupled human-natural systems. This article introduces an integrated ES modeling methodology, named ARIES (ARtificial Intelligence for Ecosystem Services), which aims to introduce improvements on these fronts. To improve conceptual detail and representation of ES dynamics, it adopts a uniform conceptualization of ES that gives equal emphasis to their production, flow and use by society, while keeping model complexity low enough to enable rapid and inexpensive assessment in many contexts and for multiple services. To improve fit to diverse application contexts, the methodology is assisted by model integration technologies that allow assembly of customized models from a growing model base. By using computer learning and reasoning, model structure may be specialized for each application context without requiring costly expertise. In this article we discuss the founding principles of ARIES - both its innovative aspects for ES science and as an example of a new strategy to support more accurate decision making in diverse application contexts.

## Introduction

The advantages of an ecosystem services (ES) view of coupled human-natural systems have been widely recognized in science, management and governance [1]. Focusing on both the biophysical mechanisms of ES provision and the socioeconomic implications of their use can allow decision makers to directly link natural capital to the societies and economies that depend on it. An ES approach can also facilitate understanding and communication of the projected consequences of resource competition in the face of scarcity as well as global and local change. Once discussions on ES became mainstream - thanks largely to the seminal Millennium Ecosystem Assessment (MEA) [2] - and lessons from many individual case studies were learned [3], a first generation of integrated, multi-ES assessment methodologies and tools has been striving to meet the needs of an audience that cuts across the academic, governmental, NGO, and corporate sectors [4], [5]. Rapid assessment methods have come to command wide interest from all these communities [5]. Yet it is generally recognized that systematic use of ES in decision- and policy-making requires a degree of accuracy that is rarely met in practice [6], [7]. Most early assessment studies [8], [9] and some recent methods [1], [10] infer ES values through production functions whose driving input is land cover type, alone or complemented by limited other structural information (e.g., vegetation type). Other methods [3] have proposed models of a more functional nature to more accurately represent the mechanistic underpinnings of ES dynamics [11]–[14].

Much less well explored remain the issues of non-linearity, incongruent scales of provision and use, thresholds, feedbacks and tipping points [15], [16] in and between the ecological and social systems that define ES dynamics [17]–. In practice, both ecological and social sciences have struggled to understand and predict changes in large and complex dynamic systems [18]–[20]. Even with adequate methodologies, the specificity and cost of assessments increase quickly with model sophistication [10], due to the need for both domain expertise and accurate time-series data for model calibration and parameterization. This makes detailed ES quantification impractical in most institutional contexts, particularly since decision making requires timely analysis [21]. Applications are further complicated by the need for true interdisciplinarity to link the underlying science and policy sides of ES and to communicate results effectively across institutional and societal boundaries. Indeed, effective translation to policy is often as challenging as the search for suitable quantification methods.

Despite these difficulties, better consideration of the dynamic aspects of ES provision is needed to understand the consequences of policy decisions impacting ES [7], [11], [12], [22]. Overlooking temporal dynamics and spatial modes of service delivery to users makes it difficult to understand and communicate *actual* values of ES accrued by societies; instead, *potential* values are commonly estimated (e.g., the amount of floodwater potentially retained by green infrastructure instead of the amount of water actually prevented from impacting flood-prone people and property). This issue may be at the source of common criticisms of overstatement of value [23] and of a subsequent lack of confidence in ES-informed policy.

Reconciling calls for simplicity and intuitiveness with the need for accuracy, specificity and dynamic resolution is challenging and risky. If on one hand decision makers (or even the scientific community) are skeptical of methods they see as complex and opaque, on the other hand the oversimplification of complex and highly diverse processes and trade-offs may yield ineffective assessments. True methodological innovation could result from incorporating enough flexibility to adapt models to diverse social, economic, and policy contexts without overly complicating their application. A previously published set of evaluative criteria for ES methods (Table 1, modified from [24]) enumerates characteristics that we believe crucial to wider consideration of ES in public- and private-sector decision making. ES assessment studies in the recent literature have begun to address these criteria, for example accounting for spatial aspects of ES dynamics [7], [12] and attempting to quantify uncertainty, e.g., using Bayesian techniques [25], [26]. Only a few of these advances have made their way into user-ready “tools” for decision-making [14], [21], [27]. The methodology proposed in this article aims to consolidate these principles by providing them with a systematic scientific foundation, so that technologies can be designed that improve their accessibility to decision makers.

**Table 1:** Evaluative criteria to improve uptake and utility of ES quantification methods in decision-making.

Criterion	Justification
Quantitative	Quantitative results are needed to compare trade-offs. Quantitative character includes providing spatially explicit results accompanied by uncertainty measures.
Time/resourcerequirements	A less time-intensive method can be more practically applied on a widespread scale.
Open source orproprietary	Methodologies delivered through open-source software and services are more transparent and can be independently applied, tested and improved.
Development anddocumentation	Methods that are well developed and documented have greater transparency and credibility, improving trust with decision makers and the public.
Scalability	Methods that can be applied across multiple spatial and temporal scales are more versatile and able to address trade-offs whose significance varies with scale.
Adaptability vs.Generality	Methods that can be applied in diverse ecological and socioeconomic contexts can be more consistently and inexpensively applied than place-specific approaches. A versatile methodology should operate with measurable accuracy across the continuum between general (low-cost, rapid assessment) and custom-tailored to specific needs and situations.
Amenability tomultiple valuationsystems	Strictly monetarily-based valuation methods are inadequate to account for all value types.

The effort described in the next sections was structured around a set of core goals. We strived first of all to improve the ES narrative so that services are consistently quantified from the viewpoint of their beneficiaries. This approach emphasizes the spatial dynamics of ES flow and use by beneficiary groups, thereby distinguishing between potential ES values and actually accrued ones. We also aimed to explicitly quantify uncertainty by modeling ES supply and demand probabilistically when appropriate. To reduce the burden of data gathering and pre-processing for the end-user, we prioritized automatic retrieval of input data from web-accessible datasets using open standards wherever possible. Finally, we devised ecoinformatics advances that support more flexible, computer-aided model assembly, including the option of transparent integration with pre-existing process models (e.g., for hydrology or nutrient dynamics) when needed input data are available and in contexts where these models have proven their effectiveness. The rest of this article discusses the first results of the 6-year, multi-investigator effort that grew from these initial goals, by addressing in each following section:

- The founding principles and technological innovations that underlie the design of ARIES.
- Results of pilot applications that exemplify both the conventional and the novel ES assessments that can be conducted with it.
- Innovations, limitations and ways forward with reference to the fast-changing ES assessment landscape.
- The positioning of ARIES in context with the criteria of Table 1, in terms of achievements and outstanding goals.

### The ARIES Methodology

The ARIES (ARtificial Intelligence for Ecosystem Services) methodology has been in development since 2007 and in use, through case studies using prototypes of evolving sophistication, since 2010. ARIES aims to quantify ES in a manner that acknowledges dynamic complexity and its consequences, but keeps its models sufficiently simple to remain tractable, general and scalable to varying levels of detail and data availability. The method is founded on two main innovations:

1. An extension of ES science intended to enrich the dominant MEA narrative with a renewed focus on beneficiaries, probabilistic analysis, and spatio-temporal dynamics of flows and scale. The result can heighten awareness of important distinctions such as that between potential and actual benefits.
2. The capability to automatically assemble the most appropriate ES models based on a library of modular components, driven by context-specific data and machine-processed ES knowledge. A model structure fitting the goals, the context and the available data can thus be used for each situation, avoiding the pitfalls of the common “one model fits all” paradigm.

The advancements made in these two research directions are interrelated, as the theoretical extensions (1) are designed to fit a modular modeling approach (2). The subsections that follow provide methodological detail on each point.

### Improving the ES Narrative: from Static Service Values to Dynamic Benefits

The MEA defined ES using a typology of “supporting,” “regulating,” “provisioning,” and “cultural” services [8], [28]–[30]. As this original classification became dominant in framing the discourse on ES, social components of ES were emphasized less strongly than ecological ones. As a result, the diversity of social values and uptake modes for the same ES in different social and geographical contexts remained relatively understudied [31]. Problems with such ecosystem-centric classifications (e.g., the potential for “double counting” of ES values [32]–[34]) prompted suggestions to shift focus to the beneficiary side [25], [35] and to better characterize the spatial locations of ES provision, beneficiaries, and spatial flows [7], [12], [22].

#### Spatially explicit benefit flows

**Figure 1:**
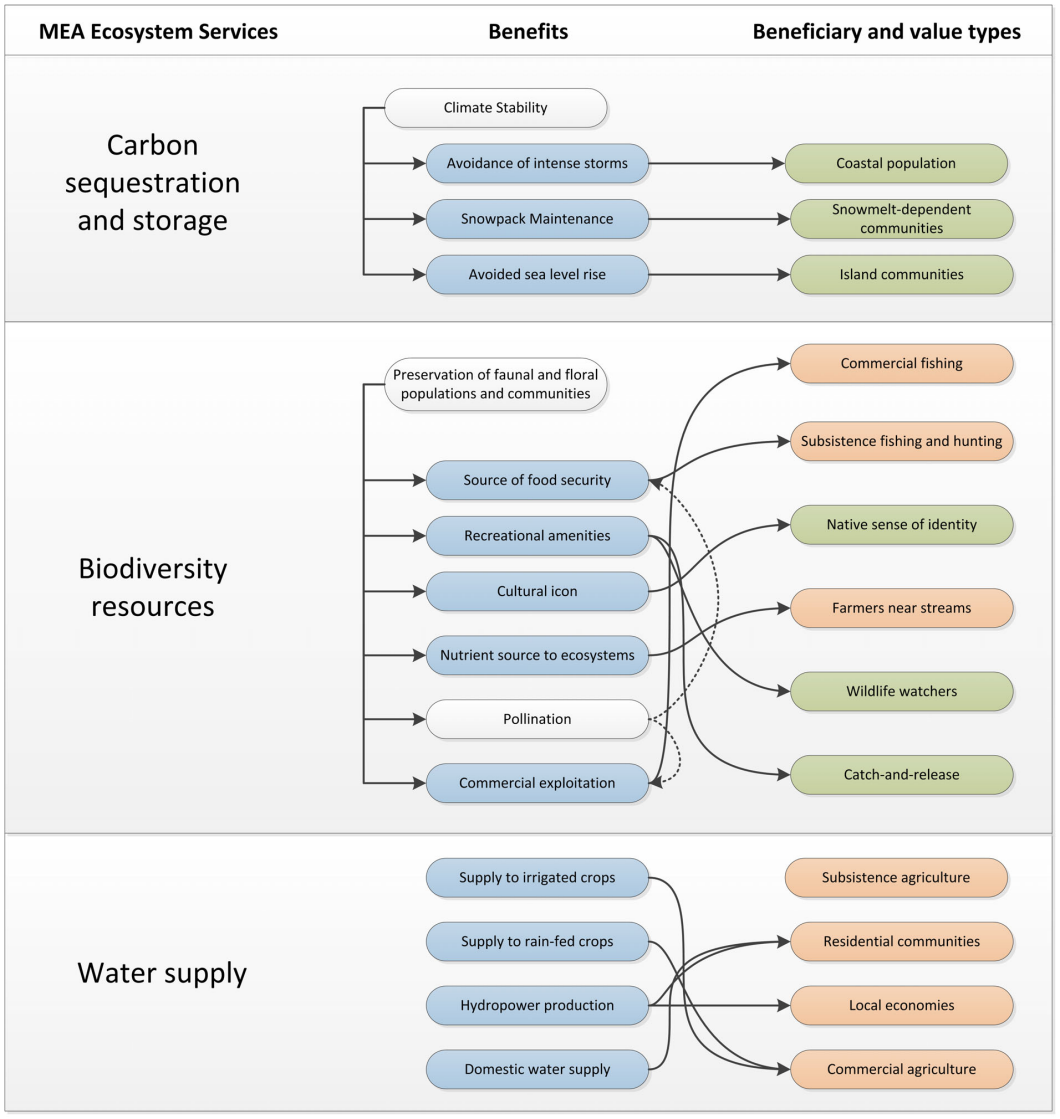
A simplified image of a small part of the ARIES knowledge base. The MEA ES categories on the left are broken down into the benefits in the middle, only some of which (in blue) are directly connected to beneficiaries. Dashed lines exemplify indirect relationships that, when taken as the description of legitimate ecosystem services, have the potential of causing “double counting” by identifying benefits that are “intermediate” and not “final”, i.e., not directly linked to beneficiaries. Beneficiaries are depicted on the right, with non-rival benefits in green and rival benefits in orange.

A redefinition of the key terms of the MEA language, now deeply ingrained in scientific and policy dialogue, would be impractical and undesirable. In order to extend the mainstream MEA conceptual model to support a systematic emphasis on beneficiaries while remaining compatible with its underlying framework, an ARIES assessment begins with the mapping of concrete and spatially explicit beneficiary groups, each uniquely characterized by their demand type and conceptualization of value (this and other key terms are defined in a glossary available as Glossary S1). We define an ES *benefit* as the outcome of the *set of processes that join a beneficiary group with specified source ecosystem(s) through a clearly identified spatio-temporal flow*. A *service* in MEA parlance corresponds conceptually to a collection of such benefits. For example, the water supply service would include separate benefits for each type of water use in an area, such as irrigation, domestic, or industrial use. Aggregate service values can then be obtained by combining the values assigned to each benefit, which may be modeled at independent spatial and temporal scales and translated into human well-being in different ways (Figure 1). Emphasizing and spatially locating beneficiaries also helps to systematically identify appropriate spatial boundaries for ES analysis. The region of interest for each benefit can be identified in space by determining the supply area capable of providing a flow of benefits that intercepts the beneficiary groups under study.

**Figure 2:**
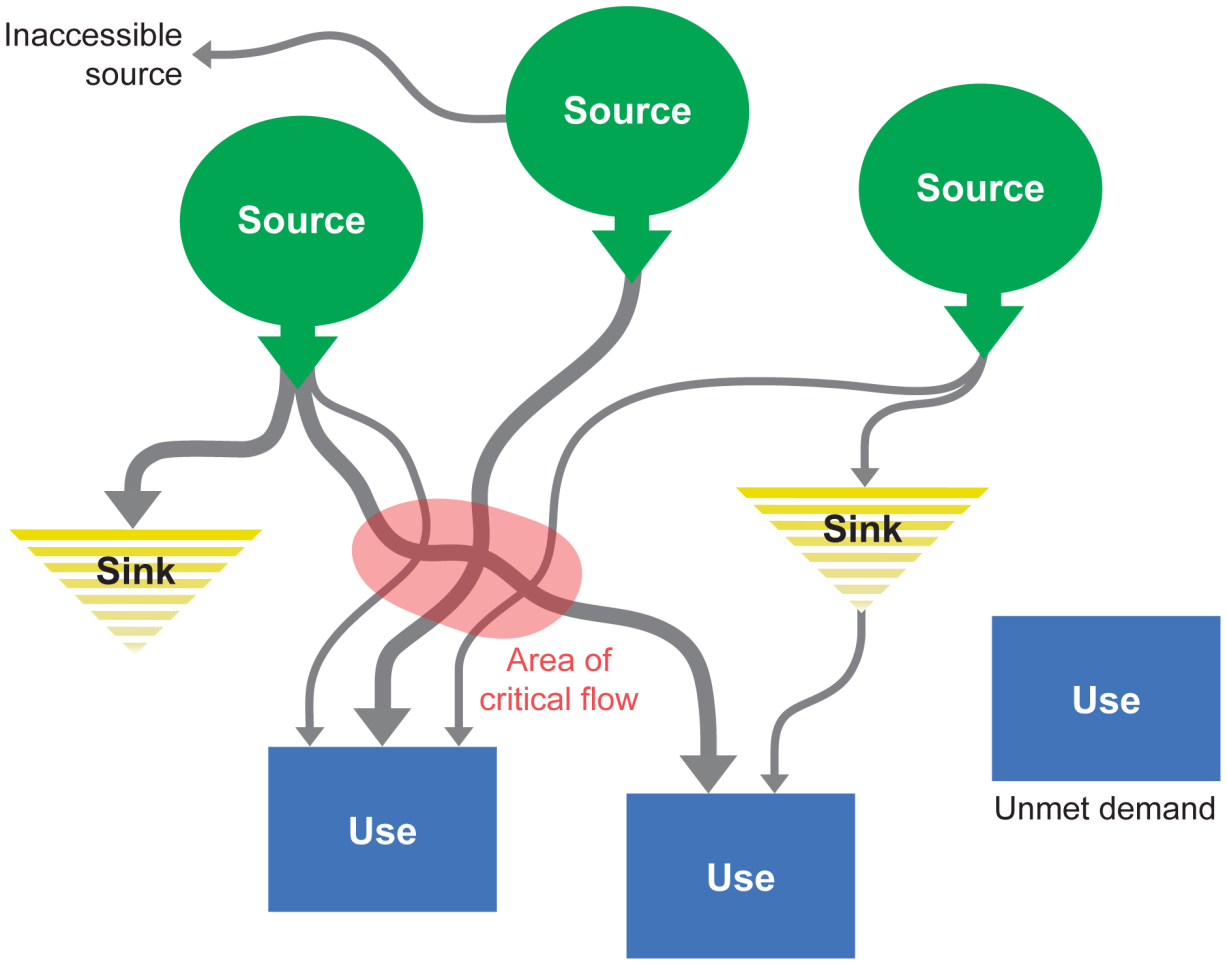
The ARIES conceptual model of ecosystem service flow dynamics.

**Emphasis on beneficiaries** allows us to improve the detail of ES models and to clarify their scale, dynamics, and eventual valuation. Once beneficiaries have been identified spatially, quantifying and mapping ES flows that reach them becomes key to distinguishing between the *potential* for benefit provision by ecosystems and the benefits *actually accrued* by society. This approach can substantially improve the accuracy of ES valuation [15], [22] and expand the value of ES assessments to decision makers [7]. In resource management scenarios, beneficiary-based maps of ES provision can be crucial for influencing management decisions that appropriately address distributional equities among “winners” and “losers” [22], [36]. Yet a mechanistic understanding of the dynamics of ES provision, use, and flow for such diverse and complex services as sediment regulation, pollination, or recreation is very challenging, and model outputs may be difficult for decision makers to understand. Like other mainstream ES models (see the review in [3]), ARIES currently uses production functions to model its main elements (source, use and sink regions: see Figure 2 and description below). Such outputs can be independently compared to those provided by other methods. However, unlike in other methods, the supply component is intended to quantify *potential* benefit provision, as it does not account for society’s use of ES.

**ES benefit transport and delivery** in time and space are handled in ARIES through dynamic *flow models*, whose algorithms use the production function output along with quantification of demand as inputs. This multi-stage approach is illustrated in Figure 2, where amounts of a *service carrier* produced in *source* (supply) regions flow to beneficiaries situated in *use* (demand) regions. Flows reach beneficiaries along physical or informational *flow paths*, which result from spatially explicit and dynamic physical processes. Demand may be rival (each user reduces the flow available for others) or non-rival (use does not appreciably reduce availability to others). The benefit connected with these flows may be *provisioning* (supplying a valuable good or service to users, such as scenic views, food, or drinking water) or *preventive* (where the contact with a biophysical flow is detrimental to human well-being, and the actual benefit is supplied by an ecosystem’s mitigation of that damaging effect, as in the mitigation of flood water, sediment, nutrients, disease, or wildfire). Along flow paths, *sink* regions may absorb or deplete the service-carrying medium – a beneficial process in the case of preventive benefits but a detrimental process for provisioning benefits. It should be noted that although the MEA ES classification uses the similar term *provisioning services*, we are not seeking to classify services when we distinguish between provisioning and preventive benefits, but instead to classify flow behaviors to enable a systematic description of how ecosystems provide benefits to people. As inputs, most ARIES flow models use the spatial distributions of the sources of service-carrying medium, beneficiary demand, and potential sinks, specified as maps covering the area under study.

The conceptual model shown in Figure 2 does not necessarily depict a spatial reality directly: regions may overlap or be remote, and, depending on the type of benefit, flows may take place in diverse ways, for example through hydrologic, informational, or transportation networks. The amount of carrier that actually reaches the beneficiaries (for provisioning benefits) or is absorbed by ecosystems on its way to beneficiaries (for preventive benefits) is the basis for assessing accrued value. Areas where the flow trajectories for one or more benefit concentrate (“area of critical ES flow” in Figure 2) can be critical to the delivery of the service even if they are not included in either source or use regions [13], [22], [27], [37].

**Values accrued by society** are, as introduced above, the result of the flow of a beneficial or detrimental carrier. The carrier may be physical (e.g., water in the case of water supply or flood regulation, CO_2_ in carbon storage and sequestration) or informational (e.g., visual information in aesthetic services). The mode of transmission and resulting spatial patterns of ES flow are determined by the nature of the carrier, the type of benefit (provisioning or preventive), the physical characteristics of the landscape, and the presence of human or natural features that act as sinks. ARIES quantifies such flows using a family of network flow propagation models [38] termed Service Path Attribution Networks (SPANs: [7], [12], [13], [26]) that simulate carrier movement, absorption and delivery for different classes of carriers (Table 2). The appropriate SPAN models are chosen and linked during model assembly depending on the benefit type.

**Table 2:** Flow characteristics for selected ecosystem services. Types are P (provisioning) or R (preventive). Rivalness is R (rival) or N (non-rival).

Service	Type	Rivalness	Benefitcarrier	Extent	Mode oftransmission	Beneficiarytypes in ARIES
Carbonsequestration &storage	R	R	CO_2_	Global	Atmosphericmixing	Greenhouse gas emitters
Riverine floodregulation	R	N	Runoff	Watershed	Hydrologicflow	Resident livesBuilt infrastructureAgricultureIndustrial assets
Coastal floodregulation	R	N	Storm surge	Coastalzone	Waverun-up	Resident livesCoastal infrastructure
Nutrientregulation	R	N	Nutrients in water	Watershed	Hydrologicflow	Commercial fishing, recreational fishing,other water-based recreation, waterfrontproperty owners
Sedimentregulation	P,R	R	Sediment	Watershed	Hydrologicflow	Farmers (P or R)Reservoirs (R)
						
Water supply	P	R	Water	Watershed	Hydrologic flow	ResidentsIndustryAgriculture
Fisheries	P	R	Fish biomass	Accessiblefisheries	Travelsimulation	Subsistence fishermen
Pollination	P	R	Pollen	Pollinatorrange	Pollinatormovement	Farmers
Aesthetic	P	N	Scenic	Viewshed	Line of sight	Property owners
value			Quality(relative ranking)			Recreational users
Open spaceproximity	P	N	Open-spacequality (relativeranking)	Accessible amenities	Human-powered access	Recreational users
Recreation	P	N	Recreationalenjoyment(relative ranking)	Recreationtravel	Travelsimulation	TourismResident users

The SPAN framework, covered in detail in Johnson et al. [12], [13], reduces the great diversity of ES benefits to a small and general taxonomy of flow types [7], based on a uniform model of connectivity and accessibility across spatial networks. This approach offers less mechanistic accuracy than could be provided by a detailed physical model, such as hydrological or sediment transport, but offers the advantages of representing all ES in a unified way and, more importantly, of being able to run in most situations with probabilistic initial conditions and manageable data requirements. The interpretation of time in SPAN models is explicit but is intentionally not temporally referenced: an initial condition progresses toward completion of an ES flow across space and time without attempting to reference the specific time when a carrier completes its traversal of a flow path. The model stops when the entire study area has been populated with flow trajectories. This approach, while more abstract and limited in predictive capabilities than a full process-based approach, facilitates spatial analysis of benefit distribution with the data available to most management situations. When data allow, the ARIES infrastructure can integrate more sophisticated models as explained in the subsection that follows.

#### Outputs of an ARIES assessment

**Figure 3:**
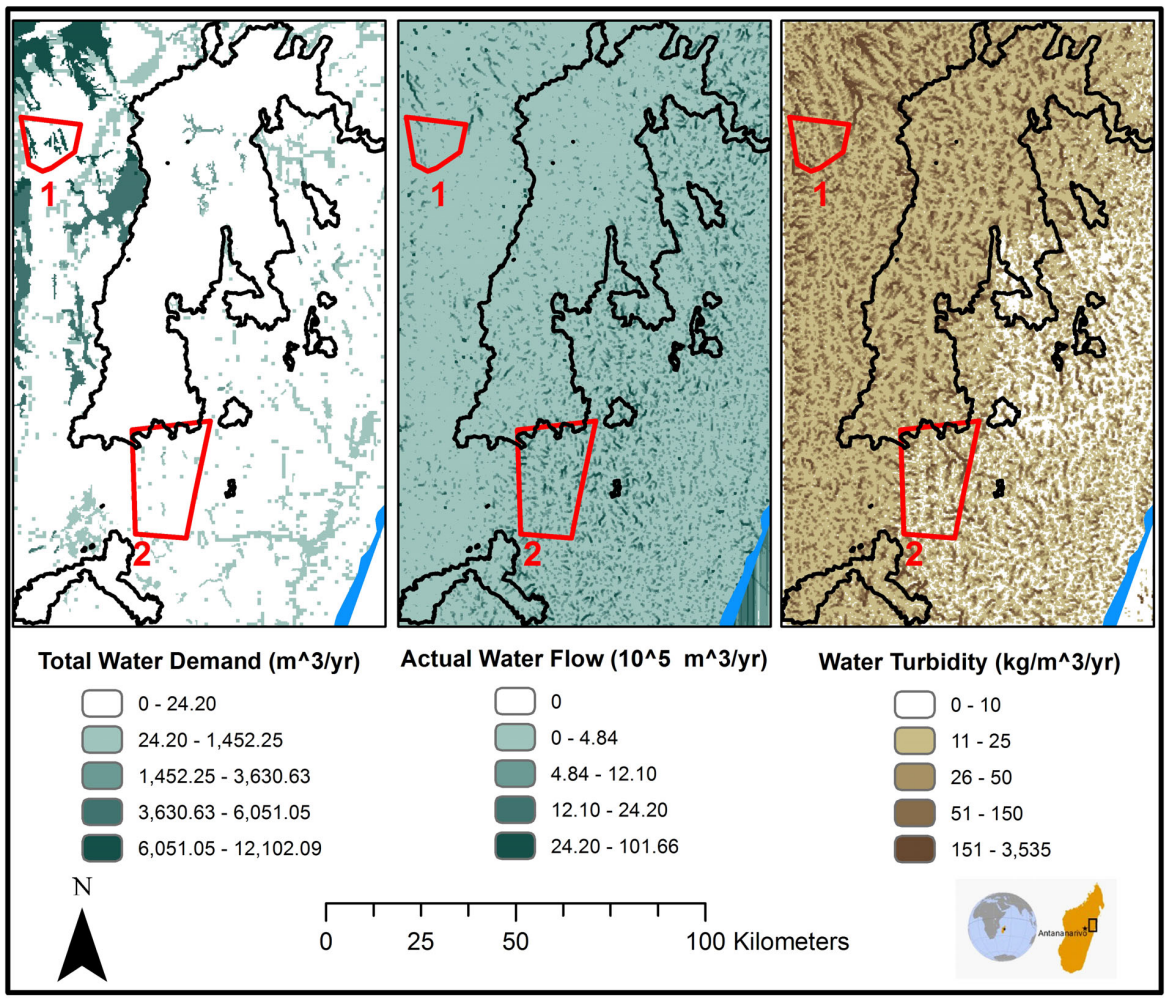
Water supply and quality in the CAZ area of Madagascar. From the left: total water demand across sectors, surface-water flow that is used by beneficiaries, and amount of sediment that is transported by hydrologic flows. Regions 1 and 2 (outlined in red) show the areas selected for comparison; the CAZ boundary is shown in black.

**Figure 4:**
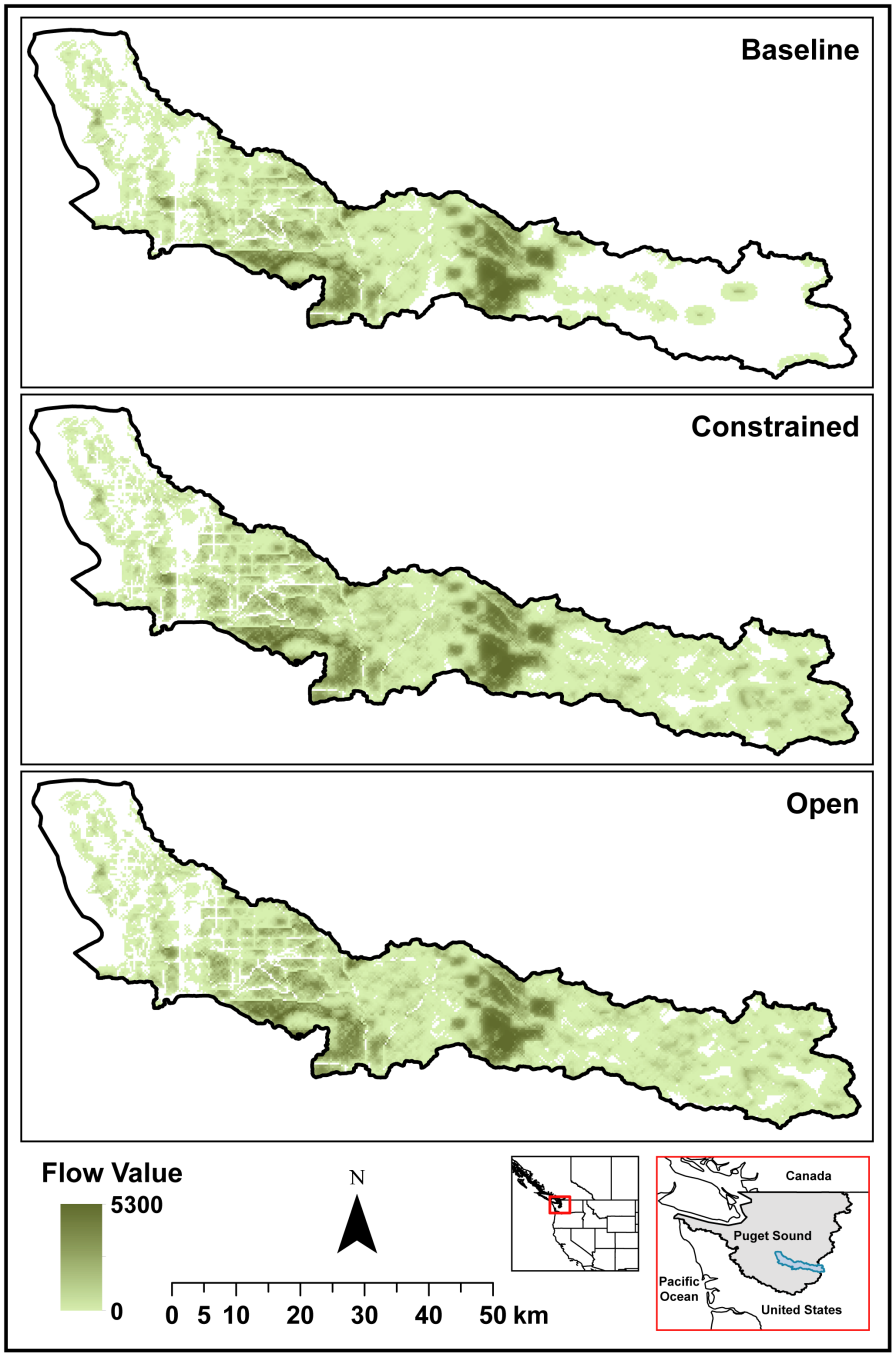
Open space proximity flows in the Green-Duwamish watershed under baseline conditions and constrained and open urban-growth scenarios. Theoretical values are in relative rankings, ranging from 0 to 100 for each cell. When multiple users have access to one source of proximity value, the value for this non-rival service is multiplied by the number of users, so total flow values can exceed 100.

The flow trajectories simulated are processed into different groups of mapped results. For provisioning benefits, a *flow density map,* displaying the amount of ecosystem benefit that has traversed each location during the course of the simulation, highlights high-value areas that are most critical to maximizing the transmission of a benefit to beneficiary groups (exemplified later on in Figures 3 and 4). For preventive benefits, flow density highlights areas where the damaging medium concentrates and can help spot areas where intervention is needed. Such maps can greatly aid planning, as in most cases it is difficult to relate flow information to either source or use areas. Because each trajectory is modeled individually, the specific amount of benefit flowing from a particular source region or to a particular beneficiary group can be determined; this can greatly aid targeted policy making. For example, targeted flow modeling could inform polluter- or beneficiary-pays policies based on service degradation or use [39].

Other decision-relevant maps produced by an analysis of benefit flows are summarized in Table 3. *Theoretical* source and sink maps show the amount of value that could be produced in ideal situations for provisioning and preventive benefits, respectively, assuming that there is a demand for all of the service produced and that the benefit flow can reach all people. *Possible* source maps show the amount that has flow paths to reach beneficiaries, but may not due to the action of sink regions. *Actual* source maps show the source of the value that actually reaches users (provisioning benefit) or of the damaging medium that actually impacts them (preventive benefit). Comparison of these maps can aid in understanding the efficiency of service delivery: for example if the possible values of a provisioning benefit are much higher than the actual, there may be room for policy-driven improvements. The objective function for scenario analysis can be the actual value accrued or another metric, for example the distributional equity of a delivered benefit.

**Table 3:** ARIES flow model outputs generated by the SPAN algorithm.

a	Definition	Estimation methods	Applications
Theoretical source, sink, use maps	*In situ* provision, depletion,or use of a service	Values calculated without the SPANmodel, not considering service flows	Understand maximum ESsupply and demandindependent of ES flow paths
Possible source, use, flow maps	Service dynamics when accountingfor flows but not sinks	Values calculated by the SPAN modelconsidering flows but not sinks	Understand ES flows in theabsence of sinks
Actual source, sink, use, flow maps	Service dynamics when accountingfor sinks and flows	Values calculated by the SPAN modelconsidering sinks and flows	Understand actual ES delivery(provisioning benefits) ordamage (preventive benefits)and values
Inaccessible source, sink, use maps	Service flows not delivered due to alack of flow connections	Calculated by subtracting actual fromtheoretical sink values and possiblefrom theoretical source and usevalues	Understand unused ES supplyor demand based oninaccessibility
Blocked source, use, flow maps	Service flows blocked by sinks	Calculated by subtracting actual frompossible values	Understand ES scarcity due tosinks in provisioning benefits,or provision of preventivebenefits, where sinks arebeneficial

Other maps link supply and demand in ways that may help spot problem areas in need of policy attention. The *blocked* source map shows the value from provisioning benefits that is produced by ecosystems but cannot get to people, because of issues such as pollution or stream diversion, or flows of preventive benefits, such as the amount of threat reduction provided to source regions by intervening landscapes (e.g., disease control, wildfire mitigation). *Inaccessible* source maps show the value that is produced by the ecosystem but cannot be accessed by people because of a lack of flow connections on the landscape. Blocked ES maps can be used, for provisioning benefits, to spot areas where intervention may be called for to restore service delivery. Inaccessible ES maps highlight areas where service production may be “underutilized”.

Result maps are produced in pairs, describing both the ecosystem sources and the human beneficiaries of each benefit. Depending on policy priorities, one or the other may be more relevant. For example, the blocked use map for water supply shows the spatial distribution of unmet demand by water users which could be met by ecosystems if benefits were not diverted to natural processes such as evapotranspiration, re-routed by infrastructure, or polluted beyond the point of usability. Conversely, the blocked source map identifies areas that produce water that is unusable for the reasons listed above. The inaccessible use map will show those areas in need that cannot be served without major structural intervention on the landscape to alter flow dynamics. For example, large water diversion projects were built over the last century to reroute previously inaccessible water to users in arid and semiarid environments. With training, a decision maker could learn to use these outputs to gain a deeper understanding of the actual service values, available policy opportunities, and location and extent of demand, both met and unmet, plus value provided for each beneficiary group [7], [26].

#### Treatment of uncertainty

To achieve the goal of making uncertainties explicit to the user, the initial conditions that enter a flow model (source, use and sink distribution) are often modeled in ARIES using a Bayesian Network (BN) approach [25], [40], [41]. This accounts for and quantifies part of the uncertainty inherent in the data and model structures that generate them. In the common occurrence of data scarcity, this also makes it possible to use informed prior probabilities gathered from local experts or prior statistical analysis [42]. The model specialization algorithms in ARIES ensure that BN models used in each context reflect available local knowledge. One practical advantage of BN models is their intuitive visual presentation, which helps decision-makers relate and contribute to the conceptual phase of model development in focus groups and participatory sessions [25], [41]. The process of *training* [42] lends BN models their data-driven character. A BN is trained to replicate correlations in trusted datasets via an iterative process that adjusts the probabilities of each outcome to best reflect the evidence seen in data. The trained BN has “learned” the correlations in the data and is more accurate in predicting the probability distributions of each outcome given the input data submitted to the model. In cases where data are missing, these distributions will exhibit greater uncertainties, maintaining in most cases some value for decision support. When source, use, or sink distributions are modeled with BNs, ARIES aims to make their interpretation intuitive by visualizing each model result as a pair of maps. Viewed side by side, they show the most likely outcome per location along with its associated level of uncertainty, computed as the coefficient of variation (for numeric predictions) or as the Shannon index of diversity (for categorical ones). Because explicit uncertainty is valuable for decision-making, the uncertainties computed in spatial BN models are carried through the flow models in the SPAN algorithms, using methods such as Monte Carlo simulation and variance propagation [26] so that flow models can also produce uncertainty information, at the expense of longer run times.

While probabilistic models do allow communication and quantification of *some* uncertainty, their linearity prevents them from incorporating and expressing feedback processes that affect natural and social systems. To partially alleviate this problem, ARIES only uses BN models to quantify initial conditions used as inputs for non-Bayesian dynamic flow models. This approach does address some of the complexity of ES flows, but of course does not completely honor the intrinsic complexity of coupled human-natural systems. In addition, the effectiveness of BN models depends on the availability of training datasets. Proper training of data-driven models can be problematic when training data are available at different resolutions and levels of reliability.

### Integrated Intelligent Modeling

A “one model fits all” approach, which relies on parameterization of a fixed model structure to accommodate differences in social and ecological contexts between case studies, is common in contemporary ES methodologies. In contexts as complex and diverse as those that characterize ES studies, the trivialization caused by such a model’s structural rigidity can compromise its utility in informing decision needs [43] and in addressing highly context-specific values and associated trade-offs [44], [45]. On the other hand, customizing model assumptions, variables and equations to match complex decision contexts usually requires great amounts of knowledge, time and expertise, an investment that is often impractical [21]. In an attempt to alleviate this near-universal limitation of modeling applications, ARIES incorporates advances in ecoinformatics that allow model structures to vary “intelligently” based on the contexts in which they are run. This is accomplished through semantic meta-modeling [46], a technique that automatically selects model components from an extensible repository reflecting data availability and the specific features of ES in each application context. Although this method is not tied to any specific conceptualization of ES (or even to ES problem space in general), the view of ES as linked, independently described source, sink and use initial conditions joined through a flow process fits an automatic model assembly method optimally by virtue of its inherent modularity.

Modularity, structural variability and structural validation of models have been hard-sought goals in modeling for decades [46]–[49]. Despite some success in the areas of model integration and synchronization [50], [51] no previously established methods were available that could directly address the needs of ARIES. The scope of the ecoinformatics innovations pioneered in ARIES, collectively termed *semantic meta-modeling* [46], [49], is three-fold:

1. **Adaptive modeling**. Model structure is not defined *a priori*, but is built for each simulation to represent the best and most problem-specific knowledge available for the context of interest. Knowledge in this sense refers to both models and data, both of which are chosen at run time by ranking the available “building blocks” for degree of fit to the context and assembling them through a process driven by artificial intelligence (termed a resolution algorithm or, for brevity, a *resolver*). Context-specific expert opinion collected and organized by ARIES modelers influences the choice of variables, algorithms, scale, and input data that determines the final model structure in each assessment. This approach relies on extensible, distributed data and model repositories made available as online services by the ARIES team and other independent research groups. Once a model has been assembled by the resolver, complete provenance information [52] is recorded, allowing a user to audit all data sources and model choices made by the system.
2. **End-user simplification**: Adoption of any technology is dependent on the simplicity of the user-side workflow. Simplifying usage without compromising accuracy and detail has been a priority in developing ARIES, in an effort to sidestep the unavoidable tension between “keeping it simple” and producing the most effective decision aid. The availability of an independently extensible model and data repository network (distributed over multiple servers) coupled with ARIES’ ability to automatically adapt data to models makes it possible to run many models without the user having to input additional data. Users need only provide data when otherwise unavailable, or to create *ex-ante* scenarios based on locally predicted changes. At the same time, the semantic validation provided by the ARIES resolver (see Model Resolution and Assembly) ensures that important factors and necessary variables are not overlooked.
3. **Independent extensibility**: The modularity of the semantic meta-modeling approach implies that multiple models can be developed for very general concepts (such as ES benefits, individually or in bundles) or more specific variables (such as soil texture or land cover). Each repository can provide data and models for any concept, and all of the available knowledge is ranked prior to selection at the assembly phase. This paradigm facilitates extension of the model base through a community process where no top-level coordination is required beyond agreement on common ontological concepts (see Model Resolution and Assembly). The knowledge base can therefore grow independently with use. Provided that knowledge repositories adhere to an agreed semantics, interoperability and consistency of the assembled models will be ensured by the model resolution process.

The main instrument to achieve these innovations is the ARIES *knowledge base*, which includes abstract concepts, models and data. In ARIES, two levels of description exist: *abstract* and *model* knowledge.

**Abstract knowledge** is composed of individual ontologies [47], [53] that organize concepts and relationships relevant to ES and biophysical modeling. Ontologies are a standardized way of representing conceptualizations as interdependent definitions of concepts and relationships [53] that can be easily extended and merged. They consist of computer-readable files that are used by widely available machine reasoning algorithms [47], [54] and remain a very active research field in artificial intelligence. ARIES’ core ontologies are based on efforts led by NASA [55] and on foundational ontologies of recognized generality [56]. In the ES-specific ontologies developed by the authors, the MEA categories are broken down into an extensible classification of ES benefits (Figure 1), which is further broken into model-relevant ecological, social and economic concepts. Each ES benefit is interpreted according to the conceptualization described in the Methods section, using source, use, sink and flow concepts for organization. An extensive analysis of case studies across multiple ES and contexts was the starting point for the assembly of these ontologies, which assist in the identification of particular beneficiaries and benefits within a target area and in model selection and assembly to best simulate their behavior.

**Model knowledge** pairs *models* to individual concepts, subject to rules that guide model selection based on specific characteristics of the application context (e.g., geographic location or the values of observations like precipitation, biome, or per capita income). In semantic meta-modeling, a model can be defined as a *strategy to observe its associated concept*, and can simply consist of a semantically annotated dataset (the preferred alternative when available) or of an algorithm of varying complexity, which may in turn require observation of other concepts in order to be computed. Models are specified in a dedicated modeling language (documented so far only in early release forms [46]) also capable of “wrapping” external models by providing semantics for their inputs and outputs, bridging to the concepts in the abstract knowledge base.

#### Model resolution and assembly

After users choose their geographical context and the concept corresponding to their ES of interest (e.g., “water supply to domestic users”), the ARIES resolver uses the abstract description of the concept to assemble the most detailed model allowed by the model knowledge (including available data). Model resolution proceeds top-down, identifying models according to the semantics of the context (e.g. a mountain watershed and a wetland region will trigger different ways of observing surface water flow), the scale of the observation (e.g. features that are only meaningful at large scales, like mountains in assessing aesthetic value, will not be included unless the region of interest is wide enough to allow their observation) and the available data (models that need input that cannot be observed will not be chosen). The resolution process builds a decision tree that resolves the principal concept to the most suitable model and, in turn, any other concepts required by the models chosen at each step, until all concepts are resolved into a computable algorithm. More than one model may be chosen for the same concept when the area of interest contains features that are different enough to require distinct model formulations in different spatial or temporal segments of the same run (e.g., if both water bodies and land areas are present). Because the model base is multi-purpose and distributed, it is common for one concept to be linked to more than one possible model; therefore it is critical to rank models by their suitability for the context. ARIES adopts a sophisticated, multiple criteria ranking algorithm that can mix objective criteria (such as spatio-temporal resolution or currency) with user-provided rankings of reliability and quality. Table 4 provides an overview of the criteria currently used for ranking. The relative weighting of these criteria is important to the outcome of model resolution: while modelers can provide customized weights on a model-by-model base, current research in ARIES is directed to devising adaptive weighting schemata that can use both objective and subjective metrics of quality of the models produced, as described briefly in the Discussion section. All other ranking criteria being equal, the algorithm prioritizes specific, detailed models that have been tagged as appropriate for the region of interest over more general, coarser alternatives, as long as data to support them exist. This integrated modeling approach supports the mixing of data-driven and hypothesis-driven models to produce the overall model structure most suited to the application context [4]. A data-driven approach such as BNs is prioritized by ARIES to compute static components, like ES production functions, wherever accepted dynamic models or the data to populate them are unavailable. A hypothesis-driven approach (such as flow models or trusted external models that have gained decision-maker confidence through repeated application and refinement) is preferred where the dynamic complexity of the phenomena (e.g., sediment or water transport) is well understood and adequate data are available for parameterization. The modeling language (named *Thinklab*) and the infrastructure implementing the semantic meta-modeling approach are open source software [57], developed concurrently with ARIES and available under the terms of the GNU General Public License [58].

**Table 4:** Current criteria for ranking model components and data selected during model assembly.

Scoring criterion	Explanation
Semantic specificity	Prioritizes data and models that are specifically defined as applying to the semantics of thecontext of interest; e.g. “carbon content in top soil layer” over more generically described“carbon soil content” when the requesting model is defined to apply to the top layer.
Scale specificity	Prioritizes data and models that are more specific for the selected spatial and/or temporalcontext, by comparing the relative proportion of coverage for the data or models with thecontext chosen for simulation.
Detail and resolution	All else being equal, data and models of higher temporal and spatial resolution will begiven priority.
Semantic distance	Data and models whose definition is closer to that of the model they are being applied to,for example by belonging to the same project or coming from related ontologies.
Currency	If no specific time period is specified for the simulation, the most current data and modelsare chosen preferentially.
User-attributed quality rankings	Users may attribute numeric ranks (0 to 100) to perceived data and model reliability.The value 50 is used if no value is specified. Other user-defined rankings can be used at thediscretion of the modeler, for example for prioritizing public data over non-disclosable onesif the model needs to be audited externally.

### Results from Application Examples

Models addressing eight ecosystem services – carbon sequestration and storage, riverine flood regulation, coastal flood regulation, aesthetic views and open space proximity, water supply, sediment regulation, subsistence fisheries, and recreation - have been developed so far using ARIES. Model components were developed and parameterized based on literature reviews and expert elicitation [27]. Efforts are underway to streamline and standardize expert elicitation procedures via surveys that can be used with future ARIES case studies. Case studies to date have focused on several locations in the USA [24], [59], Latin America, and Africa [60], [61]. Initial case study locations were selected to represent a range of ecological and socioeconomic conditions, data availability, and scientific expertise, in order to best serve as a foundation for the development of globally available models (see the Discussion section). To provide examples of early ARIES outputs, in this section we summarize key results from two contrasting case studies: water supply and quality in Eastern Madagascar and open space values in Washington State, USA. Such studies also provide examples of potential *storylines* (see the Discussion section) that users may replicate in a single action in different contexts, supported by ARIES’ automatic model building algorithms.

### Example 1: Water Quality and Quantity in Eastern Madagascar

In an integrated ecological and economic study for the World Bank WAVES (Wealth Accounting and the Valuation of Ecosystem Services) program [60], the values of ecosystem services were evaluated near a key conservation area termed the Ankeniheny-Zahamena Corridor (CAZ). ARIES flow analysis enabled comparison of water quantity and quality values for four classes of beneficiaries located within and outside a protected area. The CAZ, a largely forested, newly established protected area on Madagascar’s eastern escarpment, includes a population of nearly 350,000 people in rural communities and also supplies water to the national capital of Antananarivo. To understand the value of a protected area in providing benefits to downstream water users, we compared water budgets and erosion for an area near the CAZ but hydrologically unconnected to the protected area and with intensive agriculture (area 1) versus another adjacent and hydrologically connected to the CAZ protected area (area 2, Figure 3). Using the models from the 2011 ARIES release [27], we modeled spatially explicit water demand, simulating water-delivery dynamics when accounting for precipitation, evapotranspiration, infiltration, runoff, and rival use [60]. We then computed a preliminary water budget for the region and aggregated the spatial results to provide total figures for beneficiaries located within the two areas. Water demand for irrigation, livestock, residential consumption and tourism was estimated separately, using best practice manuals and heuristic criteria to obviate the lack of primary data for most sectors [60]. Total water demand estimates by sector are shown in Table 5.

**Table 5:** Total estimated water budget (m^3^/year) for sample areas outside (1) and adjacent to (2) CAZ.

	Total in CAZ	Sample area 1	Sample area 2
Rice agriculture	512,187,528	15,943,889	5,958,885
Non-rice agriculture	31,718,842	444,689	6,512,517
Livestock water use	684,499	206,041	54,484
Residential use	17,173,088	3,206,662	4,426,315
Annual precipitation	16,619,520,610	1,074,244,347	7,476,712,388

Erosion and sedimentation are significant environmental processes in Madagascar and strongly affect water quality [62]–[64]. To better understand the CAZ’s role in regulating sediment, a model was developed to quantify and map areas of erosion and the hydrologic flow paths of waterborne sediment. Water supply and sediment sources and sinks were quantified, which enabled comparative analysis and future valuation when economic data become available. Soil erosion was computed using a hybrid approach, automatically applied by ARIES model specialization algorithms: the Revised Universal Soil Loss Equation (RUSLE [65]) was used on gentle slopes and a probabilistic model considering soil, precipitation, and vegetation factors [27] took over when the slope was higher than 20%, too high for the RUSLE to be defensible [66]. The surface water supply and sediment transport flow models employed [27] simulate (i) the movement of surface water throughout the basins and (ii) transport and deposition of eroded sediment via hydrologic flows. Our models generated maps for which summary values of demand, supply and flow can be calculated for both sample areas and the CAZ overall (Figure 3).

To estimate the sustainability of water supply in the region, the model was run repeatedly with increasing demand levels; this allowed us to estimate approximate critical thresholds of water supply in both sample areas. Results of this analysis for the largest water use (rice agriculture) are summarized in Table 6, which shows that while current levels of demand are essentially met in both sample areas, only sample area 2 has the potential to sustain much greater demand in a future when deforestation and climate change are likely to further strain water supplies.

**Table 6:** Water supply sustainability (m^3^/year) for rice agriculture in the two areas considered.

	Sample area 1	Sample area 2
Current water need	15,943,889	5,958,885
Maximum potential	15,443,129	304,155,269
Ratio potential/need	97%	5104%

Results of the soil erosion and deposition analysis allowed us to estimate levels of sediment contamination in water, which are approximately 6 times higher outside the CAZ (area 1, 11.3 kg/m^3^.year) than adjacent to it (area 2, 1.9 kg/m^3^.year). As before, due to the great approximation and lack of primary data in all model components, these results must be considered only as comparative indicators. Still, the analysis supports the hypothesis that CAZ’s natural features are important in protecting water quality for its productive use by downstream beneficiaries, results that were previously highlighted in more in-depth studies for agricultural [67] and economic [68] productivity in other parts of Madagascar. While such results must be interpreted with caution, flow analysis enables a rapid semi-quantitative assessment of supply threats and mitigation effects that could not be obtained using other mainstream ES methodologies [3].

### Example 2: Aesthetics and Open Space Values in the Puget Sound

A case study in the Puget Sound, Washington State, USA stands in stark contrast to the previous one, in terms of both differing ecological and socioeconomic contexts and data quality and availability. The Puget Sound, the second largest estuary in the USA, is a defining social, cultural and economic feature of Washington State and home to a human population of 4.4 million, including 15 American Indian tribes and the major port cities of Seattle and Tacoma. In a recent study, we differentiated between the theoretical provision of ES (i.e., ecosystems’ capacity to supply services) and their actual delivery when accounting for the location of beneficiaries and flow paths [59]. Here we map the value provided by ecosystems to property owners via open-space proximity within the Green-Duwamish watershed, which rises from Seattle to the slopes of the Cascade Mountains (Figure 4). Although hedonic analysis can be used to monetarily value open-space proximity for property owners, spatially explicit flow models enable a comparison of theoretical and actual service delivery values under landscape change scenarios (i.e., as a result of conversion of open space to developed land use and movement of new beneficiaries into the region). To address this, we compared year 2000 (baseline) conditions to managed (constrained) and unmanaged (open) development scenarios for the year 2060 [69]. We expected widespread development to reduce theoretical open space sources that can provide value to residents, but at the same time increase the number of users and the total flow of proximity value to property owners.

Open space proximity values were mapped by identifying: 1) ecosystems providing high-quality open space (sources), 2) features that impede access to open space (sinks), and 3) housing (use) locations [27]. The values of source and sink features were ranked using a relative scale (0–100) where higher values represent the most valuable open space based on hedonic valuation studies [70], [71]. Sources, sinks and users were connected by a flow model simulating physical access to desirable spaces [27].

While the total number of new users was greater in the open development scenario, there was less high-quality open space available to provide value to property owners than in the constrained development scenario (Table 7, Figure 4). As a result, urban growth increased open space flow values, but by a greater percentage in the constrained development scenario than the open development scenario (25.7% and 23.3% increases, respectively; Table 7, Figure 4). Theoretical source values increased slightly in the development scenarios because both increase the value of open space through the designation of new parkland.

**Table 7:** Relative values for open space proximity source, use, and flows source, under alternative urban growth scenarios Green-Duwamish watershed, WA, USA.

	Constraineddevelopment	Opendevelopment
Theoretical source	+12.8%	+6.2%
Theoretical use	+16.3%	+19.4%
Actual source	+24.5%	+21.6%
Actual sink	+51.2%	+39.6%
Actual use	+24.6%	+21.8%
Actual flow	+25.7%	+23.3%

These results illustrate how placing more beneficiaries across the landscape may have the effect of increasing ecosystem service flows and, by consequence, actual values, but may simultaneously degrade the ecosystem’s underlying ability to provide the same services (i.e., theoretical values). In this case, an expansion of the urban footprint yields an increase in beneficiaries in locations where ecosystem service flows were previously inaccessible. However, land-cover change associated with new development often reduces an ecosystem’s capacity to provide services, i.e., their theoretical source values [7]. Thus a more measured approach to urban growth may actually lead to greater ecosystem service values and protection of a region’s underlying natural capital than an unconstrained development pattern.

The differences between development scenarios become far sharper when a richer set of ES and management alternatives is considered, including for example when endangered species restoration (Chinook salmon and others), flood risk reduction, retention of local farmlands, port expansion, transportation development, carbon emissions reduction, wildlife conservation, and high-tech and industrial development are all pursued in the Puget Sound Basin. Ongoing ARIES applications in the Puget Sound can be used to better quantify geographical and temporal tradeoffs in ES flows for diverse beneficiary groups.

## Discussion

In this section, we discuss the benefits and drawbacks of the different areas of innovation in ARIES and how they relate to the state of the art in spatially explicit ES modeling and valuation. We conclude by comparing our efforts to date against the evaluative criteria for ES assessment tools listed in the introduction.

### Improving Model Detail, Coverage and Transparency

The ES assessment framework proposed in this article calls for a more detailed and systematic view of ES problems than that used in many of today’s ES applications (Table 1). Yet the practical value of this new approach remains undemonstrated outside of the case studies where it has been applied so far. The value of this or other approaches to quantitative ES assessment should be seen as comparative rather than absolute; the difficulty in validation and the necessarily simple nature of the models involved make them more suitable for comparing scenario outcomes than for obtaining reliable physical estimates. Widespread testing of ES tools against discipline-specific models is still needed to understand the limitations of ES-based approaches for accurate, consistent quantification of the biophysical processes underlying ES provision [24]. Yet incorporation of dynamics and uncertainty, and the possibility of composing contextually specific models, allows for improved precision and may make the methods described suitable for more sophisticated and rigorous ES-informed decision support.

ARIES’ development has followed a bottom-up approach, starting with case studies of considerable detail conducted with partner institutions, then generalizing that knowledge to yield “global” models offering a bird’s-eye characterization of many ES in most locations, limiting data input requirements from users. The potential for large-scale adoption will clearly depend on completion of these generalized models, whose development is ongoing. These simpler models will provide a “bottom line” to which the artificial intelligence in ARIES can default, allowing it to produce results of variable detail in almost any geographic region using global data, but automatically switching to more detailed models when the knowledge base and data allow. When this stage of development is completed, ARIES will meet the needs of a larger share of potential users, growing in utility and sophistication over time. Although the integration of large model libraries poses many challenges, such libraries exist within academic institutions and agencies (such as Environmental Protection agencies or the US Army Corps of Engineers) that can be connected to ARIES. The potential of ARIES as a large-scale meta-modeling framework will be revealed as case studies and multiple ES analysis are further tested.

ARIES runs on an extensive (3.5TB of data alone at the time of this writing) and fast-growing knowledge base that includes data from both public sources and local institutions. Where data are privately owned or protected by privacy laws, the ARIES team has obtained clearance from the respective sources to release the ES assessments as derived products, as long as the original data cannot be directly downloaded or displayed. This allows public use of information that would otherwise need to be obtained by users on a case-by-case basis, adding to the up-front user investment needed to conduct an assessment. On the model side, a variety of well-known, open source physical process models are being integrated into the ARIES model base. Among these, the CAESAR-LISFLOOD flooding and erosion model [72], the PRMS water balance model [73] and a general ecosystem model broadly inspired by LPJ-GUESS [74] are being included to improve the quality of descriptions of flood, sediment, nutrient, carbon and primary production dynamics. Adding these tools will not affect the usability and complexity of the system on the user and decision-maker side, although it may produce more outputs. As a corollary of the system’s built-in flexibility, ARIES is designed to be amenable to transparent updates of the knowledge base, making it possible to perform earlier assessments repeatedly while automatically benefitting from any improved data and modeling knowledge. This also provides a mechanism for testing relative gains obtained from using complex vs. more simple models.

A precondition for effectively using ES in decision-making is acknowledging, quantifying and communicating the uncertainties that are inherent to any modeling endeavor [16]. ARIES is designed to use probabilistic initial conditions for most of its models, through the adoption of BNs, and to carry the uncertainty through the dynamic parts of its models, using methods including Monte Carlo simulation and variance propagation, so that uncertainties can be communicated to the user [26]. Importantly, only the components of overall uncertainty that relate to missing data or *known* data quality issues can be dealt with effectively in such a probabilistic model. No accounting is possible for the uncertainty that relates to the *structure* of the causal dependencies that define the Bayesian model, although this can be alleviated to some extent by adopting context-specific model assemblage rules.

Understanding strengths and limitations of these uncertainty estimates is necessary to avoid engendering a false sense of security in the decision-making process, leading users to ignore other realms of uncertainty that may have a crucial influence on outcomes in those parts of the model where the explicit uncertainty is relatively low. Like any quantitative modeling approach, BNs can lead to subjectivity and error. Guidelines and best practices for BN development have been developed [45] and are being adhered to during the ongoing development of ARIES models.

### Simplifying User Workflows

The multiple levels of detail in the ARIES model base are intended not only to produce more suitable models, but also to streamline workflows for end users, who will be able to query the system in simple ways and obtain results that automatically reflect the best available knowledge for their context. In doing so, ARIES hopes to overcome a common hurdle in ES applications today: complex usage of simple models, where models of relatively simple methodological sophistication still place a heavy burden on users for data pre-processing and parameterization. ARIES is designed to automate data choice, algorithm choice, data pre-processing and optimal attribution of levels of resolution for all ES, and to allow users to query “bundles” of ES with exactly the same workflow as single ES assessments, requesting user inputs only for knowledge that is not available. The artificial intelligence-assisted process pioneered in ARIES emphasizes user simplification without trivializing the application, a paradigm that could also be valuable for broader application in modern environmental and economic decision-making. Provenance information complements the outputs of each model run, describing data sources and model structure in the interest of transparency and traceability of problems.

To further simplify user workflow, ARIES adopts the metaphor of *storylines* to organize and present observable concepts (e.g., carbon sequestration) for the user and guide visualization after their resolution to models. Storylines connect observable concepts from the knowledge base with metadata that include explanatory descriptions of each concept, references, links, and descriptions of related case studies. Storylines are assembled automatically as models are built, documenting each model component and the rationale for its choice, and provide a blueprint for generating user-friendly documentation of results that can be viewed immediately or downloaded as digital media. ARIES is currently developed and used through a software interface geared towards modelers, which is a primary focus of current development. The intended end-user interface, however, is a web 2.0 application that allows users to search for storylines that describe concepts of interest in standard “web search” fashion, based on concept names as well as descriptions, locations, references and other metadata. Within a graphical user interface, the act of dragging and dropping one of the storyline links resulting from a search on an interactive world map specifies the geographical context and initiates model assembly and execution. The existing ARIES web interface prototype available at www.ariesonline.org [75] is, however, only demonstrational and based on an older release of the software; the described user workflow will become available in an updated application once the development of global models has been completed.

A user workflow that hides complexities under familiar metaphors and can transparently produce sophisticated models can carry subtle but important disadvantages. Early pilot tests with users have highlighted that while even limited user-level complexity is poorly tolerated by users, a lack of it can be perceived as lack of sophistication in the approach and lead to incorrect assumptions that influence decisions. To obviate this, more feedback from the modeling system can be communicated to document choices and computations as they are made, at the cost of “more output to be understood.” Further, choices made by an automated system are typically questioned by users, even if they reflect the best available expert knowledge. Incorporation of user feedback on model results may provide additional ranking criteria for model selection (Table 4), and allow a degree of “crowdsourcing” to be incorporated in the model assembly process. This can have important implications in a field where objective validation is often not an option; it is of course paramount that all subjective criteria are used in a controlled and transparent way.

### ARIES and ES Valuation

ES valuation has typically been interpreted monetarily. The realization that value is a highly multi-dimensional issue with deep ethical implications has become central in recent ES literature [76], [77]. The definition of value can vary widely in different ES applications, for example in food security [61], [78] vs. payments for ecosystem services [79], or the values reflected by indigenous communities vs. centralized governments [80]. Much as the biophysical science underlying ES provision demands careful contextualization while modeling, ES valuation cannot be trivialized with a simple definition and deserves case by case consideration within the comprehensive framework of equity [81]. The development of ARIES has so far concentrated on the biophysical modeling of ES, leaving the translation to economic value and its implications to the end user. In some circumstances, biophysical outputs (particularly the possible and actual estimates, Table 3) can be seen as representing value directly, or provide a base for inferring value beyond mere ES supply quantification [60]. Yet, the many facets of equity [81] and value [82] make the problem of case-study specific value attribution difficult to resolve in general. As a consequence, a priority in ARIES is to remain as agnostic and pluralistic as possible in its approach to valuation.

Independent of the definition, value in ARIES will ultimately be the result of a model, and the ideal representation of value for a system adopting an “intelligent” modeling approach would consist of a context-specific and customizable model of societal well-being that reflects locally established demand, customs and beliefs. If the application requires monetization, that can be accommodated via current valuation methodologies. ARIES is capable of incorporating monetization models, but no value model of this kind is currently included in the knowledge base. For generality, value is best represented as a multiple-criteria problem whose definition greatly depends on the application context [82] and target stakeholder community. Still, the need for a “common currency” to compare the effects of decisions across multiple ES and social sectors means that demand for economic estimates of ES value remains very high, no matter how controversial the surrounding issues. An often relied upon technique for estimating economic value of ES is value transfer [83], [84], based on the adaptation of primary ES valuation studies done elsewhere to a new context of interest using a set of transfer criteria [85]. Although land-cover type has often been used as the sole transfer criterion [9], function transfer – the use of mathematical functions, often derived from meta-analyses – is considered a much more robust approach [86], [87]. While ARIES does not offer benefit transfer algorithms, research on valuation is an important part of the project’s background [88]–[90] and the ARIES team is working with partners to provide bridges to valuation databases that can help generate more rigorous transfer functions [91]. For such approaches, beneficiary-based biophysical estimates of ES flows, like the actual or possible estimates computed through flow modeling [7], may assist in the development of transfer functions. This would improve on a state of the art that most commonly uses theoretical values or static proxies for these purposes.

Economic value changes in a highly nonlinear fashion in the vicinity of ecological thresholds as ES supply declines [92], [93]. Assessment methods must therefore be able to provide sufficient quantitative accuracy in assessing supply and demand, so that thresholds can be anticipated before they are encountered. Without such consideration, economic estimates should only be used when the relationship between service supply and marginal prices is known to be predictable and stable. The information on threats to supply that biophysical modeling can provide is key to the assessment of whether the linkage to economic values or prices is appropriate. For example, in the Madagascar pilot study illustrated above, supply threats have been discussed as a way to improve on the quality of the decision-making information that results from economic assessments.

### Dynamic Complexity and Trade-offs

Non-linear dynamics determine catastrophic behaviors that are of utmost interest when investigating the consequences of policies on ES delivery. They include, for example, the sudden loss of homeostatic behavior (“tipping points”) that may be encountered as particular amounts of development-induced change are reached. Despite extensive research in both ecological and social sciences on the dynamic behavior of complex systems [18], [19], our understanding of their general properties remains limited. As a result, we are unlikely to meet the various challenges related to accounting for complex human-natural system behaviors, like those listed by Carpenter et al. [16], without substantial further research. The question of how completely such non-linear dynamics can be realistically represented by ES assessment methods remains paramount. The adequacy of any model in predicting non-linear dynamics is very difficult to assess when detailed historical data are lacking, and few modeling studies exist that incorporate sufficient detail (e.g., employing a detailed and accurately calibrated hydrological assessment) to serve as a basis for qualitative cross-calibration. ARIES does account for flows using dynamic models, and the processes that underlie such flows are one of many sources of dynamic complexity. Analyzing spatially explicit and temporally referenced linkages between ecosystems and societies can provide a degree of dynamic description and the opportunity to address feedbacks, all of which can be useful for improved resource management. Yet, many other sources of ecological and social complexity remain unaccounted for when production functions are used for quantification. Agent-based models [38] that incorporate feedback on ecological systems from the societal side have begun to appear [94] and may prove useful to account for more dynamic complexity than the current state of the art in ES. The Ecosystem Services for Poverty Alleviation-Attaining Sustainable Services from Ecosystems through Trade-off Scenarios (ESPA-ASSETS) project [78] on food security and poverty alleviation through access to ES is one area where the ARIES methodology is being extended to consider such methods.

Trade-offs are ubiquitous in ES assessments and are of central importance in decision-making. Decisions can result in trade-offs between users of the same service, between different ES for the same users, or any combinations thereof, and have different meaning and relevance when considered over different space and time horizons [95]. There is currently no systematic methodology for addressing ES trade-offs, although guidelines meant for application with specific methods are appearing [96]. Systematic trade-off analysis is obviously not practical without a fully quantitative account of beneficiaries and accrued benefits. But modeling the multiple beneficiaries of a single ES can be difficult due to the collinearities resulting from rival services and societal feedbacks. An integrated approach where all such effects are modeled explicitly and simultaneously can help address the dual problem of access to and distribution of limited goods and services (“winners and losers”). For example, relatively simple outputs that hint at trade-offs can be obtained by intersecting multiple flow path outputs for a “bundle” of different ES, identifying landscape locations that are responsible for the transmission of a disproportionate amount of one or more benefit within the area of interest. Such results can, however, only be obtained if multiple ES are modeled simultaneously – i.e., subjected uniformly to the influence of each scenario and their mutual effects on each other. This is difficult with most methodologies in use today, which are typically applied separately for each service. A common limitation of current ES practice is a narrow focus on single ES, or their “bundling” without explicit regard for their inextricable interactions. While the problem is widely recognized, even comprehensive and well-funded programs (such as REDD+ [97]) remain largely focused on a single ES and only hint at the integrated perspectives.

By virtue of its computer-assisted modeling infrastructure, ARIES can produce integrated ES models with the same effort as those for single services. Land cover and other policy-controlled variables entering the models as inputs typically affect more than one service; the ARIES infrastructure ensures that a single dependency chain exists across the integrated model, so that a simulated policy intervention input affects all ES outputs. The granularity provided by ARIES in accounting for each class of beneficiaries also allows trade-offs between different stakeholders to be represented unambiguously, as each benefit and beneficiary is counted as an individual sub-model in the overall simulation.

Even with improved methodologies, important limitations remain in the face of real-life, multi-stakeholder problems. For example, the different spatial and temporal scales that accompany each conflict or policy window require careful consideration of the assumptions made in an integrated model when planning scenarios and analyzing results. The ability to quantify flow paths and address individual beneficiaries does not solve all the difficulties inherent in modeling ES trade-offs. However, such results could be used within techniques such as multiple criteria analysis [98] to assess the concordance or discordance of a set of simulated outcomes with spatially explicit or aggregated social priorities [99]. Such techniques can help minimize conflict and rank the likelihood of successful outcomes when competing policy choices must be considered. The approach can assist when defining and analyzing complex scenarios, which from the user’s point of view simply become collections of modified data or models reflecting *ex-ante* predictions, such as modified land-use data or IPCC climate-change model outputs [100]. When a particular scenario in ARIES is selected for comparison with the baseline, the data and models contained in the alternative scenario override baseline conditions that would otherwise be used to model the same concepts. This way, scenario specifications will affect all levels of the model chain, and comparing results against baseline values will highlight all trade-offs when a full ES portfolio is evaluated.

### Increasing Participation and Valuing Community Knowledge

Modularity and extendibility in modeling have two main goals: enabling a more flexible model assembly process and making it possible for independent communities to contribute knowledge that is reusable and linkable by design. ARIES was conceived from the start as a community process, where an initial model base contributed by the core team can be extended by modelers from diverse disciplinary backgrounds and application contexts. Because the knowledge and model base are theoretically unlimited in size, the system can constantly “learn” to produce better models as new data and information are accumulated. Independent case studies can reuse model components or contribute new ones to expand the model base that, given its ability to rank and switch model components, can allow the system to grow and improve with application.

Model validation through community adoption of complex paradigms is difficult and only possible over the medium- to long-term. Hopes of “viral” adoption for scientific projects in their beginnings are usually overenthusiastic at best. Yet interest in ES modeling is widespread and growing, and initiatives are in place to test this approach in practice. An intensive 2-week modeling school [101] is held annually by ARIES investigators; the models and ontologies resulting from associated workshops help address local problems brought in by participants while extending the existing model base for the benefit of future users. The first edition of the school, held in Spring 2013 [101], saw a large number of applications and the participation of thirty modelers and decision-makers from six continents with encouraging results. Preparations for the 2014 edition are currently underway. To support the growth of this community, ARIES development has prioritized the building of tools for model designers rather than model end users; the toolset used to develop and test ARIES models has been the primary focus of development, while the web 2.0 application designed for end users remains in an earlier stage.

If the goal of building a model base with extensible coverage through community participation is achieved, the role of the model-ranking algorithms that select the most context-suitable model components will become increasingly important. Just as the success of web search engines depends on the page ranking algorithms they adopt [102], mechanisms that choose the “best” model strategy can deeply affect model outcomes. This occurs despite the fact that models are merely assembled from well-tested and self-contained model components, that model choices are documented, and that ranking criteria can be inspected and modified by the user. In such cases, inclusion of crowd-sourced ratings of outputs from end users may prove useful. The current approach to ARIES capacity building, based on intensive application-driven case studies and courses focusing on ES problems of diverse nature, can also assist in developing and testing the effectiveness of different model-ranking strategies. Approaches based on careful community screening have proved very successful with relatively unstructured content (e.g., Wikipedia) but have not seen application in eliciting scientific data and models, where issues of validation are more nuanced and important. The consequences of choices in unsupervised, rule-driven model building may extend far and wide, and their full advantages and disadvantages will become clear only with continued use and development. In all cases, structural validation (both of semantic constraints through machine reasoning and by solicited community feedback on the resulting models) is expected to play a large role in the future development, use, and delivery of ARIES as a service for decision makers.

## Conclusions

Recent years have seen enormous demand for, and a growing supply of, decision maker-ready “tools” capable of quantifying ES for scenario analysis and improved decision-making [21], [103]. ARIES would most accurately be classified as a long-term research project based on a specific scientific interpretation of ES and a new modeling paradigm. Yet the method also addresses decision-makers as end users, which legitimizes the view of ARIES as another “tool on the market.” Moving a scientific target forward when potential users are so hungry for usable products is difficult, and efforts often had to be made to prevent community pressure for rapid developments from interfering with rigorous scientific thinking. The combination of a complex problem area where solutions are urgently needed but unifying theories, and even common definitions, are lacking is a serious hindrance to most applied sciences; this is particularly true for ES. In such cases, incompatible or weak assumptions may often be treated as equally legitimate and common validation criteria are difficult to identify and agree upon.

Evaluation of the current ARIES effort against the criteria we laid out in the introduction shows both met and outstanding goals. While the goal of providing quantitative, spatially explicit results that account for uncertainty is addressed extensively, there are dimensions of “quantitative” – namely those relating to predicting non-linear dynamics and its policy implications – that can only be partially met at this stage. Also, while uncertainty information can be of great value in decision-making, our pilot experiences suggest that many end users are not yet equipped to consider it in the decision-making process. Identifying the best ways to use such information and to ensure its correct interpretation at the user end remains an open problem.

In terms of resource requirements, ARIES proposes a different paradigm where user investments can be reduced drastically compared to methods that require extensive data gathering, pre-processing and supervision during model runs; yet, the achievement of this paradigm depends on complex technology that will require time to reach a level of stability that satisfies user expectations, and abundant field testing will be needed before goals of parsimony and simplicity can be definitively achieved. The issue of open source and verifiability has similar corollaries: ARIES’ modeling platform and model base are both distributed under an open source license and every model is fully documented, but the novelty of the approach, use of BNs, and the considerably more expansive vision of ES emerging from ARIES outputs may yield initial impressions of opacity for some users. Areas where we believe ARIES can bring important innovations to the ES field are those of scalability and generalizability, due to the structural variability built into the modeling approach. This is of course contingent on satisfactorily addressing the novel challenges posed by the growth of a dynamic, modular model base. With respect to valuation, ARIES is currently not addressing the definition of “currencies” for ES results, either economic or non-economic, and relies on end users to interpret biophysical flow results as they see most appropriate. As integration proceeds with models that can more directly express value, such as those based on multiple-criteria analysis and value transfer, it will be easier to directly assess the added value of ARIES in meeting demand for valuation outputs.

We believe that proper accounting for ES is one of the key scientific challenges for the new millennium, and that finding space for return-free intellectual development in this field can be as important as delivering methods to decision makers in the short term. The end goal of ARIES is both to seed new discussion in the science behind ES and to pioneer innovations in technology that can allow this science to become more readily usable to inform sustainable development. Important hurdles remain in achieving a vision as ambitious as the one described here. Yet, the field of ES has so far seen very simplified approaches aimed towards rapid assessment and quick policy advice. No current ES modeling effort has attempted to fully account for a coupled human-natural system dynamics that can only be investigated with in-depth and long-term scientific study. Indeed, the complex and multiple-scale modeling required for such assessments is likely to remain impossible or impractical, at least on a routine basis, for some time. Yet, with increased availability of remotely sensed data and low-cost computing power, more refined instruments to assist decision-making even in data- and resource-limited policy contexts become increasingly practical, and it is our hope that this work contributes to the scientific and policy discourse surrounding ES and their values.

## Supporting Information

Glossary S1 Glossary of concepts to support ecosystem service flow quantification in ARIES.
(DOC)
