# Supplementary material for: A Methodology for Adaptable and Robust Ecosystem Services Assessment
Source: PLoS One. 2014 Mar 13;9(3):e91001. doi: 10.1371/journal.pone.0091001 (PMC3953216; doi:10.1371/journal.pone.0091001)
Supplement: Glossary S1 — Glossary of concepts to support ecosystem service flow quantification in ARIES. (DOC) [file pone.0091001.s001.doc]

**S1**. Glossary of concepts to support ecosystem service flow quantification in ARIES.

| **Concept** | **Definition** | **Purpose for flow modeling** |
| --- | --- | --- |
| Benefits-based approach to ecosystem services modeling | Concrete, unique, and final beneficiaries of ecosystem services | Avoids double counting, supports spatially explicit mapping and valuation of beneficiaries |
| Ecosystem service carrier | A mobile matter, energy, or information quantity represented in physical units or relative rankings | Used in SPAN to track the route and quantity of the service flow between source, sink, use locations |
| Provisioning benefit | Benefits provided by sources and flow paths, where interaction with the carrier is beneficial to users | Defines sources as valuable and sinks as detrimental regions |
| Preventive benefit | Benefits provided by a sink, where interaction with the carrier is detrimental to users | Defines sources as detrimental and sinks as valuable regions |
| Rivalness | Indicates whether service use does or does not deplete available quantity for other users | Rival use depletes the carrier weight available for “downstream” users; non-rival use does not |
| Limited or unlimited source, sink, use behavior | Source, sink, or use locations have either finite or infinite capacity to provide, deplete, or use a service | Determines whether source, sink, and use locations have limited or unlimited capacity to provide, deplete, or use a service |
| Flow routing type | Services move via specific routes (e.g., hydrologic or transportation networks, lines of sight, distance decay) | Determines the routes that carriers follow within the SPAN model |
| Source region | A location that supplies a carrier | Sources generate carrier agents for subsequent flow simulation |
| Sink region | A location that depletes the quantity of a carrier available for future use | Sinks deplete the carrier available for “downstream” users |
| Use region | The location of users – specific human beneficiary groups – on the landscape | Users benefit from or are damaged by interaction with a carrier |
| Flows | The spatially explicit routing of an ecosystem service from sources to users | Quantified and mapped flows, a major output of the SPAN model |
| Theoretical source, sink, use maps | *In situ* provision, depletion, or use of a service | Values calculated by the SPAN model without considering flows. For source, corresponds conceptually to “potential” values computed by other mainstream methods. |
| Possible source, use, flow density maps | Service dynamics when accounting for flows but not sinks | Values calculated by the SPAN model without considering sinks |
| Actual source, sink, use, flow density maps | Service dynamics when accounting for sinks and flows | Values calculated by the SPAN model considering sinks and flows |
| Inaccessible source, sink, use maps | Service flows not delivered due to a lack of flow connections | Calculated by subtracting possible from theoretical values |
| Blocked source, use, flow density maps | Service flows blocked by sinks | Calculated by subtracting actual from possible values |
